# The Metabolism of Separase Inhibitor Sepin-1 in Human, Mouse, and Rat Liver Microsomes

**DOI:** 10.3389/fphar.2018.00313

**Published:** 2018-05-07

**Authors:** Feng Li, Nenggang Zhang, Siddharth Gorantla, Scott R. Gilbertson, Debananda Pati

**Affiliations:** ^1^Center for Drug Discovery, Baylor College of Medicine, Houston, TX, United States; ^2^Department of Molecular and Cellular Biology, Baylor College of Medicine, Houston, TX, United States; ^3^Advance Technology Core, Baylor College of Medicine, Houston, TX, United States; ^4^Texas Children’s Cancer Center, Houston, TX, United States; ^5^Department of Pediatrics, Baylor College of Medicine, Houston, TX, United States; ^6^Department of Chemistry, University of Houston, Houston, TX, United States

**Keywords:** separase inhibitor, liver microsomes, sepin-1 metabolism, CYP450 inhibition, metabolite identification

## Abstract

Separase, a known oncogene, is widely overexpressed in numerous human tumors of breast, bone, brain, blood, and prostate. Separase is an emerging target for cancer therapy, and separase enzymatic inhibitors such as sepin-1 are currently being developed to treat separase-overexpressed tumors. Drug metabolism plays a critical role in the efficacy and safety of drug development, as well as possible drug–drug interactions. In this study, we investigated the *in vitro* metabolism of sepin-1 in human, mouse, and rat liver microsomes (RLM) using metabolomic approaches. In human liver microsomes (HLM), we identified seven metabolites including one cysteine–sepin-1 adduct and one glutathione–sepin-1 adduct. All the sepin-1 metabolites in HLM were also found in both mouse and RLM. Using recombinant CYP450 isoenzymes, we demonstrated that multiple enzymes contributed to the metabolism of sepin-1, including CYP2D6 and CYP3A4 as the major metabolizing enzymes. Inhibitory effects of sepin-1 on seven major CYP450s were also evaluated using the corresponding substrates recommended by the US Food and Drug Administration. Our studies indicated that sepin-1 moderately inhibits CYP1A2, CYP2C19, and CYP3A4 with IC_50_ < 10 μM but weakly inhibits CYP2B6, CYP2C8/9, and CYP2D6 with IC_50_ > 10 μM. This information can be used to optimize the structures of sepin-1 for more suitable pharmacological properties and to predict the possible sepin-1 interactions with other chemotherapeutic drugs.

## Introduction

Separase is an enzyme that resolves chromosomal cohesion and centriole engagement during mitosis. It is a cysteine protease in the CD clan (Uhlmann et al., 2000), with a catalytic domain structure similar to that of caspase (Viadiu et al., 2005; Winter et al., 2015; Lin et al., 2016). Although the N-terminus of separase varies, its C-terminus containing the proteolytic-active site is conserved from yeast to humans (Zhang and Pati, 2017). The canonical role of separase in the cell cycle is to cleave cohesin Rad21 at the onset of anaphase to separate sister chromatids. Separase is also required for centrosome duplication by cleaving centrosomal Rad21 (Nakamura et al., 2009; Tsou et al., 2009; Schockel et al., 2011) and pericentrin/kendrin (Lee and Rhee, 2012; Matsuo et al., 2012) to disengage centrioles. In addition, separase is involved in DNA damage repair (Nagao et al., 2004; McAleenan et al., 2013) and vesicle trafficking (Bembenek et al., 2007; Richie et al., 2011; Moschou et al., 2013, 2016; Bai and Bembenek, 2017). Due to its importance in numerous cell processes, the activity of separate is tightly regulated (Ciosk et al., 1998; Zou et al., 1999; Stemmann et al., 2001; Gorr et al., 2005; Holland and Taylor, 2006). Notably, separase is overexpressed in many human cancers of breast, bone, brain, blood, and prostate (Pati, 2008; Meyer et al., 2009; Mukherjee et al., 2014a,b; Zhang and Pati, 2017). Overexpression of separase induces aneuploidy and tumorigenesis in mouse models (Zhang et al., 2008; Mukherjee et al., 2014b). Using a high-throughput screen, we have identified a novel small molecular inhibitor of separase, named sepin-1, which inhibits separase activity in a non-competitive way (Zhang et al., 2014). Sepin-1 selectively inhibits the growth of cancer cell lines including breast cancer, leukemia, and neuroblastoma. It also inhibits the growth of breast cancer xenografts in mice. Sepin-1 induces apoptosis and its effect on the inhibition of cell growth is positively correlated to the level of separase in the cancer cells and tumors (Zhang et al., 2014). It suggests that sepin-1 possesses a great potential to be used for cancer treatment, particularly to treat separase-overexpressed tumors. Additionally, the use of inhibitors could resolve the non-canonical functions of separase as well.

In drug development, a drug’s metabolism plays a critical role in its efficacy and safety. As a preclinical evaluation of sepin-1, here we have profiled the Phase I metabolism and bioactivation of sepin-1 in HLM (Uhlmann et al., 2000), MLM, and RLM, using metabolomic approaches, which have been shown to be powerful tools for studying drug metabolism (Li et al., 2014; Liu et al., 2015, 2016). The metabolic enzymes contributing to the metabolism of sepin-1 were identified using recombinant CYP450s, and the possible reactive metabolites were investigated in HLM using glutathione (GSH) as the trapping agent. The inhibitory effects of sepin-1 on seven common CYP450s were also evaluated. These results can be used further to optimize the structures of sepin-1, resulting in more suitable pharmacological properties and reduced possible toxicity, as well as to predict the metabolism-mediated possible interactions of sepin-1 with other chemotherapeutic drugs.

## Materials and Methods

### Materials

Sepin-1 (2,2-dimethyl-5-nitro-2H-benzimidazole-1,3-dioxide) with >97% purity was purchased from ChemBridge (San Diego, CA, United States). Phenacetin, efavirenz, paclitaxel, (*S*)-mephenytoin, quercetin, α-naphthoflavone, ticlopidine, ketoconazole, sulfaphenazole, and dextromethorphan were purchased from Cayman Chemical (Ann Arbor, MI, United States). Reduced GSH, diclofenac sodium, quinidine, midazolam solution, formic acid, and NADPH were obtained from Sigma-Aldrich (St. Louis, MO, United States). HLM, MLM, and RLM, and the recombinant human CYP450s (EasyCYP Bactosomes) were purchased from XenoTech (Lenexa, KS, United States). All the solvents for LC and MS were of the highest grade commercially available.

### Metabolism of Sepin-1 in HLM, MLM, RLM, and Recombinant CYP450s

Incubations were conducted in 1× phosphate-buffered saline (1× PBS, pH 7.4), containing 30 μM sepin-1, 1.0 mg HLM, MLM, RLM, or 2 pmol of each cDNA-expressed P450 enzyme (control, CYP1A2, 2A6, 2B6, 2C8, 2C9, 2C19, 2D6, 2E1, and CYP3A4) in a final volume of 190 μl. After 5 min of pre-incubation at 37°C, the reaction was initiated by adding 10 μl of 20 mM NADPH (final concentration 1.0 mM) and continued for 30 min, with shaking at 37°C. Incubations lacking NADPH served as controls. Reactions were terminated with 200 μl of ice-cold methanol, followed by vortexing for 30 s and centrifuging at 15,000 × *g* for 15 min. Each supernatant was transferred to an auto sampler vial, and 5.0 μl was injected on to UHPLC coupled with a QTOFMS system for metabolite analysis. Incubations were conducted in quadruplicate for HLM, in triplicate for MLM and RLM, and in duplicate for cDNA-expressed P450 enzymes.

### Trapping Reactive Metabolites Using Glutathione

The reactive metabolites were trapped with GSH in our current study. The experiments were conducted in 1× PBS (pH 7.4), containing 30 μM sepin-1, 1.0 mg HLM, and GSH (2.5 mM) in a final volume of 190 μl. After 5 min of pre-incubation at 37°C, the reactions were initiated by the addition of 10 μl of 20 mM NADPH (final concentration 1.0 mM) and continued for 30 min with gentle shaking. Incubations in the absence of NADPH and trapping agents were used as controls. The reactions were quenched by adding 200 μl of ice-cold methanol. The mixtures were vortexed for one 30 s and centrifuged at 15,000 × *g* for 15 min. The supernatants were transferred to sample vials for analysis. Incubations were performed in triplicate.

### Inhibition of Sepin-1 on CYP450s

Incubations were performed in 1× PBS (pH 7.4), containing 0, 0.156, 0.312, 0.625, 1.25, 2.5, 5, 10, 20, or 40 μM sepin-1, 2 pmol of each cDNA-expressed P450 enzymes, and corresponding substrates: CYP1A2 (phenacetin, 40 μM, 20 min incubation), 2B6 (efavirenz, 20 μM, 30 min), 2C8 (paclitaxel, 10 μM, 30 min), 2C9 (diclofenac, 5 μM, 15 min), 2C19 [(*S*)-mephenytoin, 40 μM, 20 min], 2D6 (dextromethorphan, 5 μM, 15 min), and CYP3A4 (midazolam, 3 μM, 10 min) in a final volume of 190 μl. After 5 min of pre-incubation at 37°C, the reaction was initiated by adding 10 μl of 20 mM NADPH (final concentration 1.0 mM) and continued for a specific time as presented above with gentle shaking. Incubations without sepin-1 were used as controls. Reactions were terminated by the addition of 200 μl of ice-cold methanol, vortexing for 30 s, and centrifuging at 15,000 × *g* for 15 min. Each supernatant was transferred to an auto sampler vial and 5.0 μl was injected on to UHPLC coupled with a QQQMS system for the specific metabolite analysis. A MRM method was used. Incubations were conducted in duplicate. Positive controls were performed by using a known specific inhibitor for each of the isoform assays (Supplementary Table S1).

### UHPLC–QTOFMS Analyses

The separation of sepin-1 and its metabolites was achieved using a 1260 Infinity Binary LC System (Agilent Technologies, Santa Clara, CA, United States) equipped with 100 mm × 2.1 mm (Agilent XDB C18) column. The column temperature was maintained at 40°C. The flow rate was 0.3 ml/min, with a gradient ranging from 2% to 98% aqueous acetonitrile containing 0.1% formic acid in a 15-min run. QTOFMS was operated in a positive mode with electrospray ionization. Ultra-high pure nitrogen was applied as the drying gas (12 l/min) and the collision gas. The drying gas temperature was set at 325°C, and the nebulizer pressure was kept at 35 psi. The capillary voltages were set at 3.5 kV. During MS, real-time mass correction and accurate mass were achieved by continuously measuring standard reference ions at *m/z* 121.0508 and 922.0098 in the positive mode. The MS/MS of sepin-1 metabolites was performed in a targeted mode with a default isolation width of *m/z* 4 and collision energy ramp ranging from 10 to 45 V.

### UHPLC–QQQMS Analyses

The separations of each enzyme substrate and its specific metabolite were achieved using a 1260 Infinity Binary LC System (Agilent Technologies, Santa Clara, CA, United States) equipped with the 50 mm × 4.6 mm (Agilent XDB C18). The flow rate was 0.3 ml/min, and the mobile phases were water and acetonitrile with 0.1% formic acid. QQQMS was operated in a positive mode with electrospray ionization. Ultra-high pure nitrogen was applied as the drying gas (14 l/min) and the collision gas. The drying gas temperature was set at 280°C and the nebulizer pressure was kept at 20 psi. The capillary voltages were set at 3.6 kV for positive mode and 3.0 kV for negative mode. The MRM transitions for the specific metabolites are listed in **Table 1**. The second transitions were used for confirmation purposes.

**Table 1:** Summary of metabolites of sepin-1 in liver microsomes.

RT (min)	Observed *m/z* [M+H]	Calculated *m/z* [M+H]	Mass error (ppm)	Predicted molecular formula	Metabolite ID	Source
4.25	224.0675	224.0671	1.8	C_9_H_10_N_3_O_4_	Sepin-1	ND
4.78	194.0929	194.0930	-0.5	C_9_H_12_N_3_O_2_	M1	HLM, MLM, RLM
1.98	194.0927	194.0930	-1.5	C_9_H_12_N_3_O_2_	M2	HLM, MLM, RLM
6.51	385.1618	385.1624	-1.6	C_18_H_20_N_6_O_4_	M3	HLM, MLM, RLM
5.82	385.1613	385.1624	-2.8	C_18_H_20_N_6_O_4_	M4	HLM, MLM, RLM
6.50	385.1613	385.1624	-2.8	C_18_H_20_N_6_O_4_	M5	HLM, MLM, RLM
3.24	313.0961	313.0971	-3.2	C_12_H_16_N_4_O_4_S	M6	HLM, MLM, RLM
1.75	499.1608	499.1611	-0.6	C_19_H_26_N_6_O_8_S	M7	HLM, MLM, RLM


*ND, not detected.*

### Data Analysis

Mass chromatograms and mass spectra were acquired by MassHunter Workstation Data Acquisition Software (Agilent, Santa Clara, CA, United States) in centroid and profile formats from *m/z* 100 to 1000. The acquisition rate was set as 1.5 spectra per second. Centroid and integrated mass chromatographic data were processed by Mass Profinder and Mass Profiler Professional Software (Agilent, Santa Clara, CA, United States) to generate a multivariate data matrix. The corresponding data matrices were then exported into SIMCA13 (Umetrics, Kinnelon, NJ, United States) for multivariate data analysis. OPLS-DA was conducted on Pareto-scaled data. For the QQQMS data, QQQ Quantitative Analysis Software (Agilent, Santa Clara, CA, United States) was used for metabolite analysis. For chemometric analysis, matrix data were processed from *m/z* 50 to 600. The experimental data are presented as mean ± SEM. Statistical differences between two groups were determined by Student’s *t*-test.

## Results

### Profiling of Sepin-1 Metabolism in HLM Using a Metabolomic Approach

**FIGURE 1:**
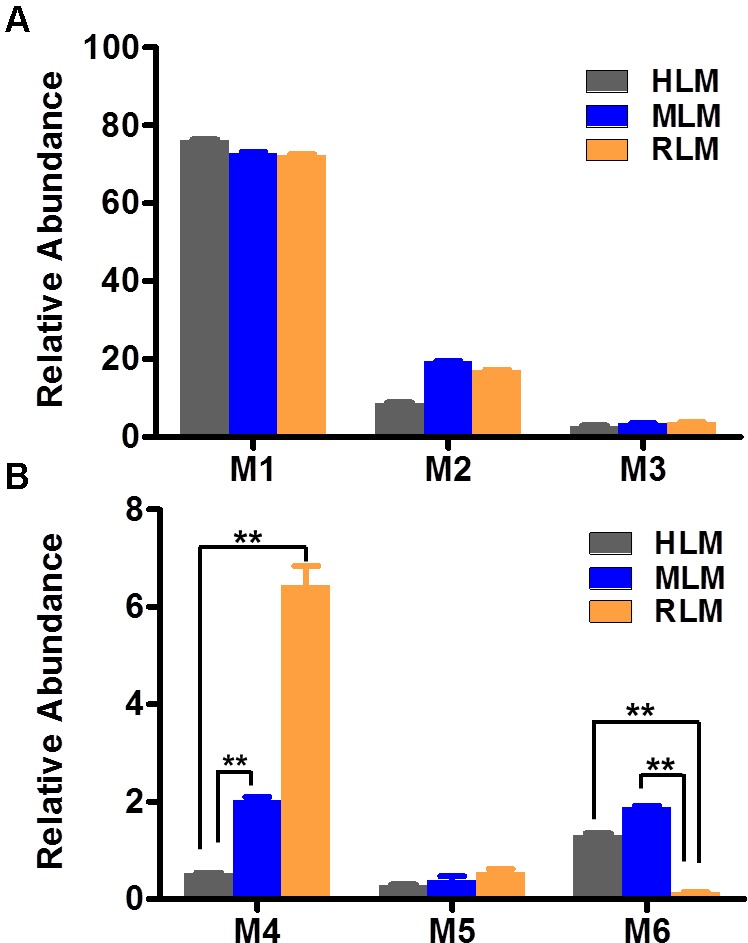
Relative abundance of metabolites in human, mouse, and RLM (HLM, MLM, and RLM), respectively. Quadruplicate incubations for HLM and triplicate incubations for MLM and RLM were carried out in 1× PBS (pH 7.4) containing sepin-1 (30 μM), HLM (1.0 mg/ml), MLM (1.0 mg/ml), or RLM, with or without NADPH (1.0 mM). The metabolites were measured by UHPLC–QTOFMS. The overall abundance of metabolites was set as 100% in each sample. The data are expressed as mean and SEM (*n* = 3 or 4). **(A)** Relative abundance of major metabolites, M1–M3. **(B)** Relative abundance of minor metabolites, M4–M6. ^∗∗^*p* < 0.01.

The results of metabolomic analysis on the ions generated from UHPLC–QTOFMS analysis of control and sepin-1 group are shown in Supplementary Figure S1. Metabolomic analysis unraveled two clusters (Supplementary Figure S1A) corresponding to the control and sepin-1 group in the score plots, which indicated the chemical components are different between control and sepin-1 groups. The S-plot generated from OPLS-DA displays ion contribution to group separation in HLM (Supplementary Figure S1B). The top ranking ions were identified as sepin-1 metabolites, which were marked with M1-6 in the S-plot (Supplementary Figure S1B). The majority of the sepin-1 metabolites in HLM were also found in MLM and RLM (**Figure 1**). The information associated with sepin-1 metabolites is summarized in **Table 1**. The relative abundance of metabolites in HLM, MLM, and RLM is presented in **Figure 1**. Overall, seven sepin-1 metabolites and adducts, including one cysteine–sepin-1 adduct (M6, Cys–sepin-1) and one GSH–sepin-1 adduct (M7, GSH–sepin-1), were identified in HLM.

### Identification of Reduced Metabolites (M1 and M2) in Sepin-1 Metabolism

**FIGURE 2:**
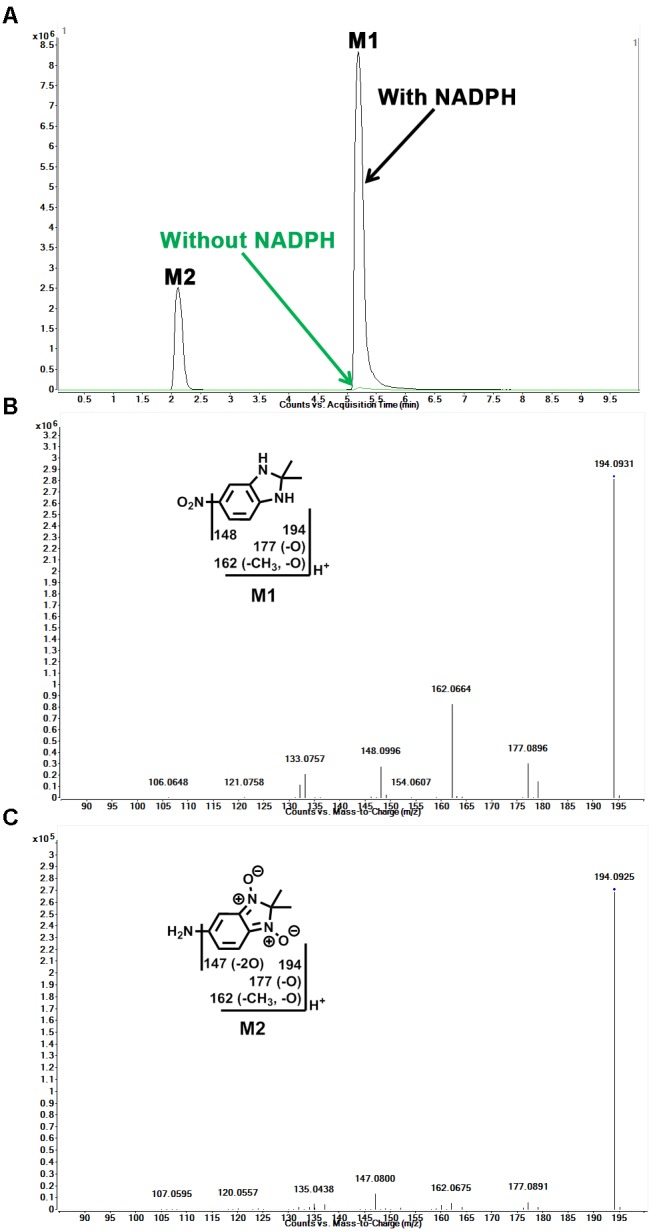
Identification of reduced metabolites (M1 and M2) in HLM. Incubation of sepin-1 was conducted as described in **Figure 1**. The metabolites were analyzed using UHPLC–QTOFMS. Structural elucidations were performed based on accurate mass measurement (mass errors <10 ppm) and MS/MS fragmentations. MS/MS fragmentation was conducted with collision energy ramping from 10 to 45 V. The major daughter ions are interpreted in the inlaid structural diagrams. **(A)** The chromatograms of metabolites M1 and M2. **(B)** The MS/MS of M1. **(C)** The MS/MS of M2.

In the incubation of sepin-1 in HLM, two major reduced metabolites (M1 and M2) were observed and characterized (**Figure 2**). The formation of metabolites M1 and M2 is NADPH dependent (**Figure 2A**). M1 was eluted at 4.78 min (**Figure 2A**), having a protonated molecule [M+H] at *m/z* = 194 Da (**Figure 2B**). The MS/MS of M1 produced the major fragment ions at *m/z* 177, 162, and 148, and the fragment ions have been interpreted in the inlaid structural diagram (**Figure 2B**). The structure of M1 was confirmed by comparison with the retention time and MS/MS fragment pattern of a synthesized standard compound (Supplementary Figure S3). M2 was eluted at 1.98 min (**Figure 2A**), having a protonated molecule [M+H] at *m/z* = 194 (**Figure 2C**). The MS/MS of M2 produced the major fragment ions at *m/z* 177, 162, and 147. The fragmental ions have been interpreted in the inlaid structural diagram (**Figure 2C**).

### Identification of Dimer Metabolites (M3, M4, and M5) in Sepin-1 Metabolism

**FIGURE 3:**
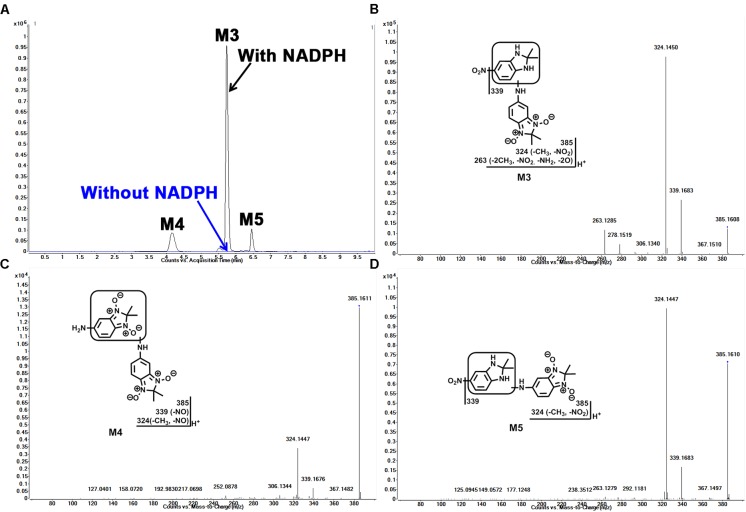
Identification of dimer metabolites (M3, M4, and M5) in HLM. Quadruplicate incubations were performed in 1× PBS (pH 7.4) containing sepin-1 (30 μM), HLM (1.0 mg/ml), with or without NADPH (1.0 mM). The metabolites were analyzed using UHPLC–QTOFMS. The metabolite elucidation conditions are described in **Figure 2**. **(A)** Chromatograms of M3, M4, and M5. **(B)** MS/MS of M3. **(C)** MS/MS of M4. **(D)** MS/MS of M5.

In our study, we found dimer metabolites (M3–M5) formed in HLM, MLM, and RLM. Metabolite M4 is the most abundant dimer in RLM (**Figure 1B**). The dimer formation is NADPH dependent. The formation of dimer metabolites indicated that molecular interaction occurred post-metabolism. The dimer metabolites M3, M4, and M5 were identified by MS/MS and their accurate masses. M3, eluted at 5.82 min (**Figure 3A**), had a protonated molecule [M+H]^+^ at *m/z* = 385. The fragmental ions at *m/z* 339 suggested denitration. The other fragmental ions at *m/z* 324 and 263 are interpreted in the inlaid structural diagram (**Figure 3B**). M4, eluted at 4.25 min (**Figure 3A**), had a protonated molecule [M+H]^+^at *m/z* = 385. The fragmental ions at *m/z* 339 and 324 have been interpreted in the inlaid structural diagram (**Figure 3C**). M5, eluted at 6.50 min (**Figure 3A**), also had a protonated molecule [M+H]^+^ at *m/z* = 385. The fragmental ions at *m/z* 339 suggested that denitration has occurred. The other fragmental ion at *m/z* 324 is interpreted in the inlaid structural diagram (**Figure 3D**).

### Identification of Cys–Sepin-1 Adduct (M6) and GSH–Sepin-1 Adduct (M7) in Sepin-1 Metabolism

**FIGURE 4:**
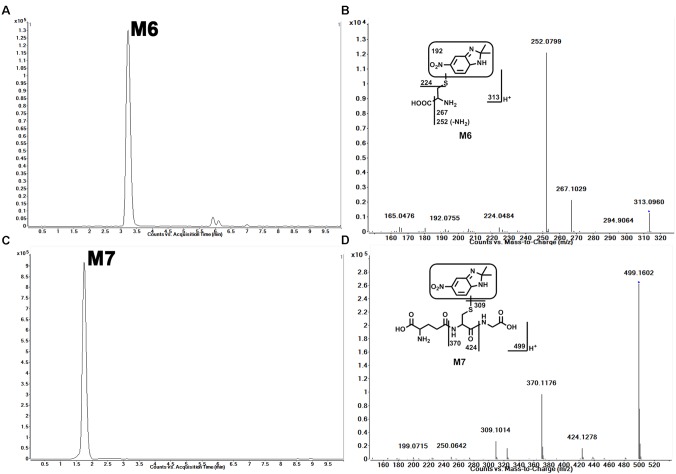
Identification of Cys–sepin-1 adduct (M6) and GSH–sepin-1 adduct (M7). Triplicate incubations were conducted in 1× PBS (pH 7.4) containing sepin-1 (30 μM), HLM (1.0 mg/ml), with or without GSH (2.5 mM), and NADPH (1.0 mM). The metabolites were analyzed using UHPLC–QTOFMS. The metabolite elucidation conditions are described in **Figure 2**. **(A)** Chromatogram of M6. **(B)** MS/MS of M6. **(C)** Chromatogram of M7. **(D)** MS/MS of M7.

In HLM, we observed the formation of Cys–sepin-1 (M6). The conjugation of sepin-1 with cysteine suggested that sepin-1 could be bioactivated to form the reactive metabolites in the incubation system. In HLM, in the presence of GSH, we observed GSH–sepin-1 adduct (M7) as expected and identified it with its accurate mass by MS/MS. Our studies suggest that the formation of Cys–sepin-1 and GSH–sepin-1 adducts is NADPH dependent (data not shown). M6, eluted at 3.24 min (**Figure 4A**), had a protonated molecule [M+H]^+^ at *m/z* = 313. The fragmental ions of M6 at *m/z* 267, 252, 224, and 192 were interpreted in the inlaid structural diagram (**Figure 4B**). M7, eluted at 1.75 min (**Figure 4C**), had a protonated molecule [M+H]^+^ at *m/z* = 499. The major fragment ion of M7 at *m/z* 424, 370, and 309 is interpreted in the inlaid structural diagram (**Figure 4D**).

### The Role of CYP450s in Sepin-1 Metabolism

Incubation of sepin-1 with different human cDNA-expressed P450s (control, CYP1A2, 2A6, 2B6, 2C8, 2C9, 2C19, 2D6, 2E1, and CYP3A4) revealed that multiple enzymes are involved in the formation of M1–M5 (**Table 2**). CYP3A and CYP2D6 are primary enzymes contributing to the formation of metabolites M2–M5. The formation of M1 is almost equally mediated by different enzyme isoforms (**Table 2**).

**Table 2:** Role of P450s in the metabolism of sepin-1.

	M1	M2	M3	M4	M5
Control	79.5	41.5	17.0	20.3	19.0
CYP1A2	86.3	50.5	36.8	40.2	16.5
CYP2A6	90.8	76.1	51.0	59.6	14.5
CYP2B6	91.9	85.9	59.0	55.0	19.1
CYP2C8	91.3	63.6	52.0	53.5	28.2
CYP2C9	90.4	57.2	53.0	51.4	24.5
CYP2C19	90.8	67.5	49.1	60.4	23.4
CYP2D6	96.9	100.0	95.5	100.0	20.3
CYP2E1	89.9	53.2	46.8	55.6	28.4
CYP3A4	100.0	95.3	100.0	97.1	100.0


*cDNA-expressed P450s were used to determine the role of individual CYP450 in sepin-1 metabolism. The incubation conditions of sepin-1 were detailed in experimental procedures. All samples were analyzed by UHPLC–QTOFMS. The peak area of each metabolite from the incubation with the highest abundance was set as 100%. All data are expressed as mean (*n* = 2).*

### Inhibitory Effect of Sepin-1 on CYP450s

**FIGURE 5:**
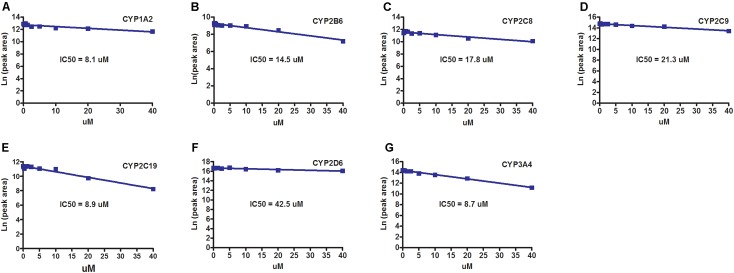
The inhibitory effects of sepin-1 on CYP450s. Incubations were performed in 1× PBS (pH 7.4), containing 0, 0.156, 0.312, 0.625, 1.25, 2.5, 5, 10, 20, or 40 μM sepin-1, 2 pmol of each cDNA-expressed P450 enzymes, and corresponding substrates. The specific metabolites were analyzed using UHPLC–QQQMS. **(A)** Inhibition of CYP1A2. **(B)** Inhibition of CYP2B6. **(C)** Inhibition of CYP2C8. **(D)** Inhibition of CYP2C9. **(E)** Inhibition of CYP2C19. **(F)** Inhibition of CYP2D6. **(G)** Inhibition of CYP3A4.

The inhibitory effects of sepin-1 on seven main CYP450 isoforms (CYP1A2, 2B6, 2C8, 2C9, 2C19, 2D6, and 3A4) were evaluated. The specific metabolites, acetaminophen for CYP1A2, 8-*O*-efavirenz for CYP2B6, 6-*O*-paclitaxel for CYP2C8, 4-*O*-diclofenac for 2C9, 4-*O*-mephenytoin for 2C19, dextromethorphan *O*-demethylation for 2D6, and 1-*O*-midazolam for 3A4, were monitored by LC–MS/MS. Our data suggested that the IC_50_s of sepin-1 on CYP1A2 (8.1 μM), CYP2C19 (8.9 μM), and CYP3A4 (8.7 μM) are less than 10 μM (**Figures 5A,E,G**), whereas IC_50_s on CYP2B6 (14.5 μM), CYP2C8 (17.8 μM), CYP2C9 (21.3 μM), and CYP2D6 (42.5 μM) are greater than 10 μM (**Figures 5B–D,F**).

## Discussion

In this study, we employed a LC–MS-based metabolomic approach to profile sepin-1 (a potent separase inhibitor and an investigational chemotherapeutic drug candidate) metabolism and bioactivation in HLM, MLM, and RLM. Metabolomic approaches could readily screen out the sepin-1-related metabolites and reactive intermediates from the biomatrix (Li et al., 2011, 2012). We identified seven sepin-1 metabolites and adducts, including reduced metabolites M1 and M2, dimer metabolites M3–M5, and cysteine–sepin-1 adduct (Cys–sepin-1) M6 and GSH–sepin-1 adduct M7, using the metabolomic technology. All the metabolites were presented in HLM, MLM, and RLM. M4 was the most abundant dimer in RLM, but only a trace amount was observed in HLM. However, the Cys–sepin-1 adduct M6 was mainly formed in HLM and MLM (**Figure 1B**). M1 and M2 are dominant metabolites in all the three species of HLM, MLM, and RLM (**Figure 1**). Among these metabolites, one Cys–sepin-1 adduct (M6) was detected and characterized. The formation of M6 was also NADPH dependent. In HLM, sepin-1 is metabolized rapidly to the dominant-reduced metabolites M1 and M2. Therefore, the most abundant metabolite M1 was synthesized (Do et al., 2016) and the inhibitory activity of M1 on separase will be assayed next.

The reactive metabolites play a critical role in the pathogenesis of idiosyncratic adverse drug reactions (Attia, 2010; Park et al., 2011; Thompson et al., 2016). The formation of Cys–sepin-1 adduct (M6) indicated that sepin-1 could be bioactivated to generate the reactive metabolite in HLM. The mechanism of the formation of Cys–sepin-1 adduct in HLM is not known yet, but the adduct M6 is derived from the major metabolite M1 by conjugating with cysteine in HLM. Reduced GSH is an abundant physiological nucleophile and frequently used to capture soft electrophiles (e.g., epoxides, quinones, quinone imines, and quinone methides) in *in vitro* systems (Evans et al., 2004). Our study revealed one GSH–sepin-1 adduct (M7) in the incubations of sepin-1 in HLM with GSH. Although the mechanisms of formation of GSH–sepin-1 adducts in HLM are not clear, the formation of GSH–sepin-1 adduct further suggested that the sepin-1 could produce the reactive electrophiles in HLM. Generally, excess reactive electrophiles are capable of covalent binding to protein, DNA, and other biomolecules. It is thought that cellular function is compromised in certain cases, followed by organ toxicity (O’Brien et al., 2005). These reactive metabolites from sepin-1 may induce some adverse effects. In drug development, researchers attempt to reduce the risk factors of toxicity by minimizing the formation of reactive metabolites. This information will be helpful for medicinal chemists to further optimize the structure of sepin-1 to improve its safety. For example, medicinal chemists could decrease the formation of reactive metabolites by blocking their metabolic sites via the structural modifications.

The mechanisms of sepin-1 reduction (M1 and M2) and dimer formation (M3–M5) are not clear yet. The liver microsomes involved in the formation of reduced metabolite is known (Leskovac and Popovic, 1980; Placidi et al., 1993; Wang et al., 2005). Our study suggested that multiple P450s enzymes contribute to sepin-1 metabolism. CYP3A4 and CYP2D6 are the primary enzymes responsible for the generation of metabolites M2–M5 (**Table 2**). For M1, all the test isoenzymes make similar contributions to the formation of M1. Because CYP3A4 is the most abundant drug-metabolizing enzyme in the liver, the formation of M1 should be mainly mediated by CYP3A4. The role of CYP3A4 in the formation of M1 can be investigated further using Cyp3a-null mice. As the GSH–sepin-1 adduct was formed by GSH conjugating with the major metabolite M1, the formation of GSH–sepin-1 could have been mediated by multiple P450 enzymes as well. Other enzymes (e.g., cytochrome b5 reductase in the microsomes) have been reported to be implicated in the reduction of *N*-oxide (Zheng et al., 2011). In addition, the reduction of *N*-oxide (e.g., quinoxaline-1,4-dioxide) could be mediated by aldehyde oxidase and xanthine oxidase in the cytosol as well (Zheng et al., 2011; Mu et al., 2014). The exact role of P450s and reductases in the sepin-1 reduction will be further investigated using CYPP450 inhibitor (1H-benzotriazol-1-amine and carbon monoxide) and the liver microsomes from CYP450 reductase-knock out mice. Non-enzymatic reduction of *N*-oxide by heme of P450 will be tested using the boiled microsomes as well in the future study (Takekawa et al., 2001).

As discussed as above, sepin-1 could be metabolized by multiple enzymes, other drugs thus may have little effect on the metabolism of sepin-1, when sepin-1 is co-administrated. However, sepin-1 may have significant effects on the metabolism of co-administrated drugs and cause drug–drug interactions by inhibiting their major drug-metabolizing enzymes. In the current study, the inhibitory effects of sepin-1 on seven main CYP450 isoenzymes (CYP1A2, 2B6, 2C8, 2C9, 2C19, 2D6, and 3A4) were evaluated. These seven enzymes metabolize >90% of marked drugs. Typically, inhibition potency of a compound can be categorized into three classification bands: (1) potent inhibition with IC_50_ < 1 μM; (2) moderate inhibition with 1 μM < IC_50_ < 10 μM; or (3) no or weak inhibition with IC_50_ > 10 μM [Food and Drug Administration (FDA) draft guide for industry, 2012]. Using the corresponding substrates recommended by FDA and monitoring their specific metabolites by LC–MS/MS, we unraveled that sepin-1 has moderate inhibition on CYP1A2 (IC_50_ = 8.1 μM), CYP2C19 (IC_50_ = 8.9 μM), and CYP3A4 (IC_50_ = 8.7 μM). Our data also indicate that sepin-1 has weak inhibition on CYP 2B6 (IC_50_ = 14.5 μM), CYP2C8 (IC_50_ = 17.8 μM), CYP2C9 (IC_50_ = 21.3 μM), and CYP2D6 (IC_50_ = 42.5 μM). These data suggest that sepin-1 may have some moderate interaction with the drugs mainly metabolized by CYP1A2, 2C19, and/or CYP3A4. For example, GEF, a tyrosine kinase inhibitor, is mainly metabolized by CYP3A4 (Liu et al., 2015). The pharmacokinetics of GEF may be moderately altered by sepin-1 if GEF is taken together with sepin-1. In the current study, we only evaluated the co-incubation of sepin-1 and enzyme substrates to evaluate the inhibitory effects of sepin-1. The metabolism-/mechanism-based inhibition was not performed here.

**FIGURE 6:**
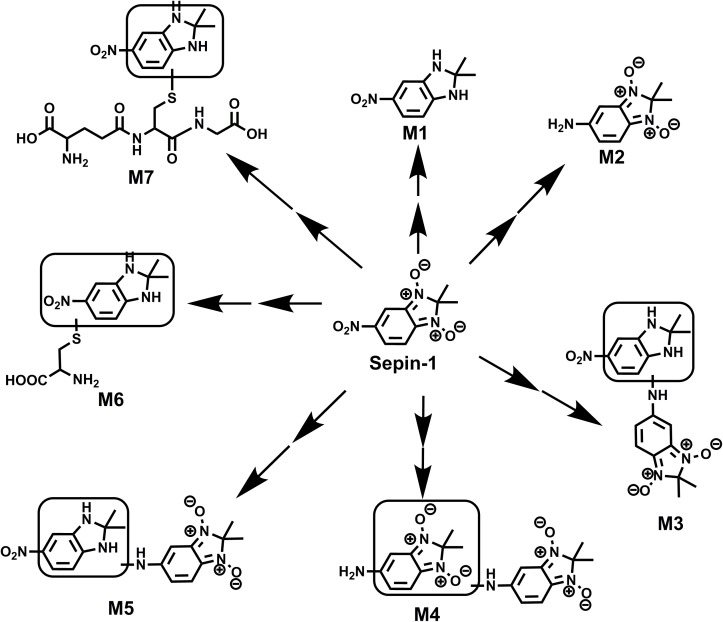
Summary of putative structures of GEF metabolites and adducts. All structures were determined based on the exact mass (mass error <10 ppm) and MS/MS fragments.

In summary, the metabolism of sepin-1 in HLM, MLM, and RLM was extensively studied using LC–MS-based metabolomic approaches. This study identified a total of seven metabolites and adducts related to sepin-1 (**Figure 6**) including Cys–sepin-1 and GSH–sepin-1 adducts. The enzyme contributing to the formation of sepin-1 metabolism was identified using recombinant P450 isoenzymes. Additionally, the inhibitory effects of sepin-1 on the seven main CYP450 isoenzymes were evaluated. This study provides the foundation for optimizing the structures and predicting the possible drug–drug interactions from the metabolic prospective of sepin-1, our lead separase inhibitor for cancer therapy. Further studies are suggested to illustrate the role of other enzymes contributing to reduced metabolites of sepin-1, metabolism of sepin-1 *in vivo*, and the inductive effects of sepin-1 on CYP450 isoenzymes *in vitro* and *in vivo*.

## Author Contributions

DP and FL participated in the research design. NZ, SG, SRG, and FL conducted the experiments. FL performed the data analysis. DP, NZ, and FL wrote or contributed to the writing of the manuscript.

## Conflict of Interest Statement

The authors declare that the research was conducted in the absence of any commercial or financial relationships that could be construed as a potential conflict of interest.

## Abbreviations

CYP450: cytochrome P450
GEF: gefitinib
HLM: human liver microsomes
MLM: mouse liver microsomes
MRM: multiple-reaction monitoring
NADPH: nicotinamide adenine dinucleotide phosphate
OPLS-DA: orthogonal projection to latent structures-discriminant analysis
QQQMS: triple quadrupole mass spectrometry
QTOFMS: quadrupole time-of-flight mass spectrometry
RLM: rat liver microsomes
UHPLC: ultra-high performance liquid chromatography
