# Supplementary material for: The Metabolism of Separase Inhibitor Sepin-1 in Human, Mouse, and Rat Liver Microsomes
Source: Front Pharmacol. 2018 May 7;9:313. doi: 10.3389/fphar.2018.00313 (PMC5949348; doi:10.3389/fphar.2018.00313)
Supplement: Supplementary file 1 [file Presentation_1.PDF]

The Metabolism of Separase Inhibitor, Sepin-1 in the Human, Mouse, and Rat Liver Microsomes

Feng Li*[2,3,4], Nenggang Zhang[1], Siddharth Gorantla[1], Scott R. Gilbertson[5], Debananda Pati*[1,2,3]

[1]Texas Children's Cancer Center, and Department of Pediatrics, Baylor College of Medicine, Houston, TX, USA

[2]Center for Drug Discovery, Baylor College of Medicine, Houston, TX 77030, USA

[3]Department of Molecular and Cellular Biology, [4]Advance Technology Core, Baylor College of Medicine, Houston, TX 77030, USA

[5]Department of Chemistry, University of Houston, Houston, Texas 77204, USA

Corresponding authors: Dr. Feng Li, Center for Drug Discovery, Advance Technology Core, Department of Molecular and Cellular Biology, Baylor College of Medicine, Houston, TX 77030, USA Phone: (713) 798-3623; Email: fl3@bcm.edu

Dr. Debananda Pati, Texas Children's Cancer Center, and Department of Pediatrics, Baylor College of Medicine, Houston, TX, USA

Phone: 832-824-4575; Email: pati@bcm.edu

Supplemental Figure 1. Metabolomic analysis of control group and sepin-1 group in the incubations with human liver microsomes.

Supplemental Figure 2. The chromatogram of Sepin-1 and MS/MS.

Supplemental Figure 3. The chromatograms of M1 and synthetic standard.

Supplemental Table 1. Summary of substrates, specific metabolites, transition ions, and positive control inhibitors used in the inhibitory experiment of sepin-1 on P450s

S_Fig. 1. Metabolomic analysis of control group and sepin-1 group in the incubations with human liver microsomes.

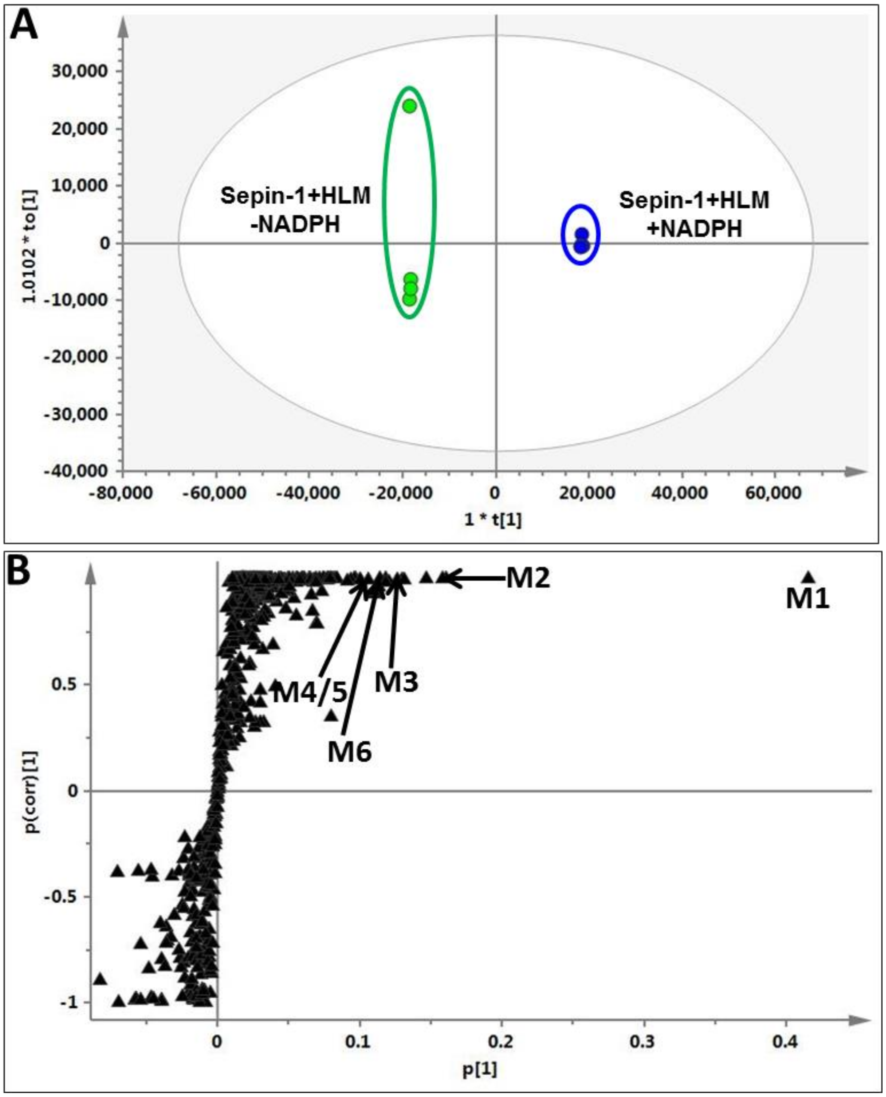

The incubation conditions of sepin-1 were detailed in experimental procedures. All samples were analyzed by UHPLC-TOFMS. A, separation of control and sepin-1 group in OPLS-DA score plot. The t[1] and to[1] values represent the score of each sample in principal component 1 and 2, respectively. B, loading *S*-plot generated by OPLS-DA analysis. The *x*-axis is a measure of the relative abundance of ions, and the *y*-axis is a measure of the correlation of each ion to the model. The top ranking ions are labeled. The number of ions (metabolite identification) shown is in accord with Table 1.

S_Fig. 2. The chromatogram of Sepin-1 and MS/MS.

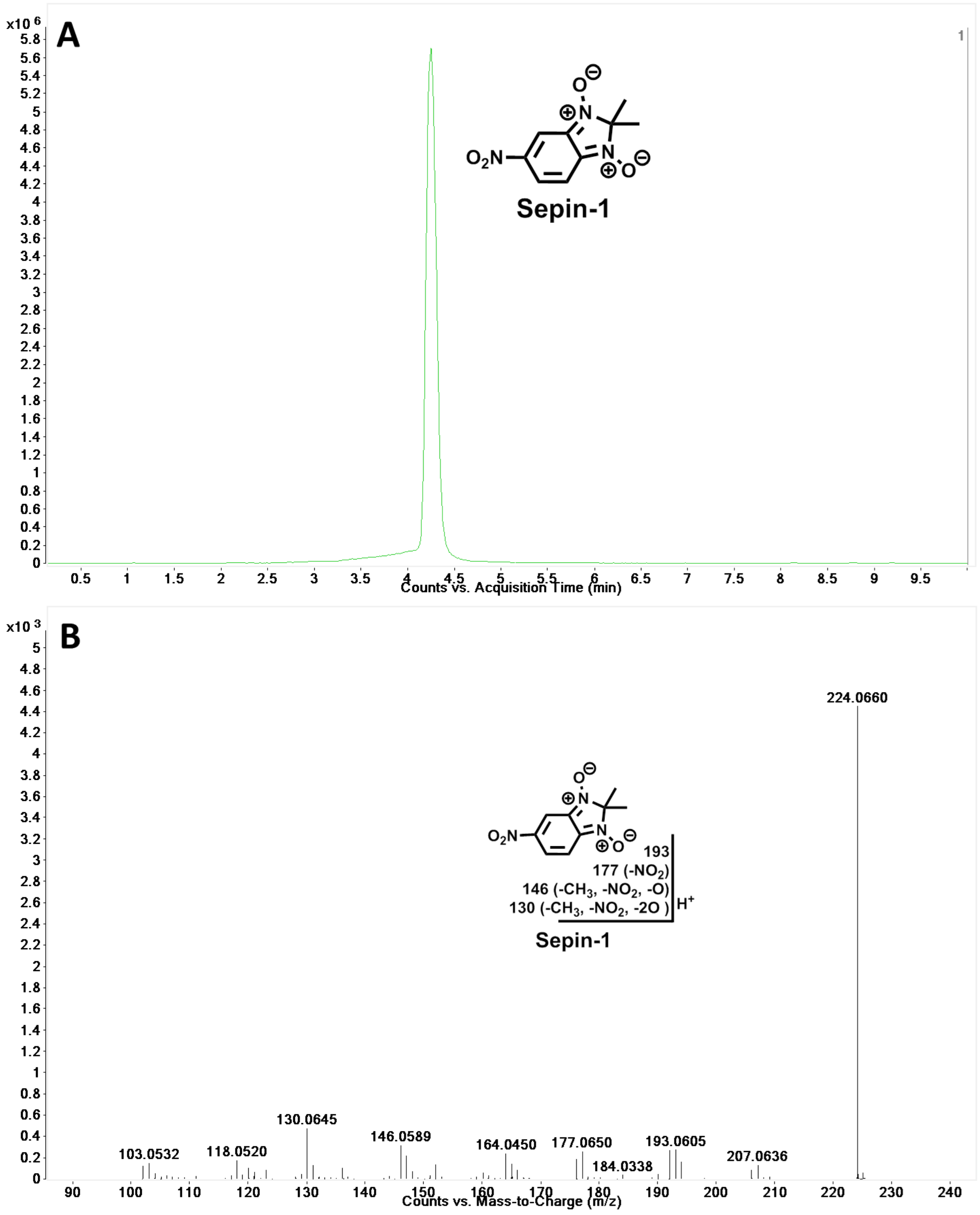

(**A**) The chromatograms of Sepin-1. (**B**) The MS/MS of Sepin-1.

S_Fig. 3. The chromatograms of M1 and synthetic standard

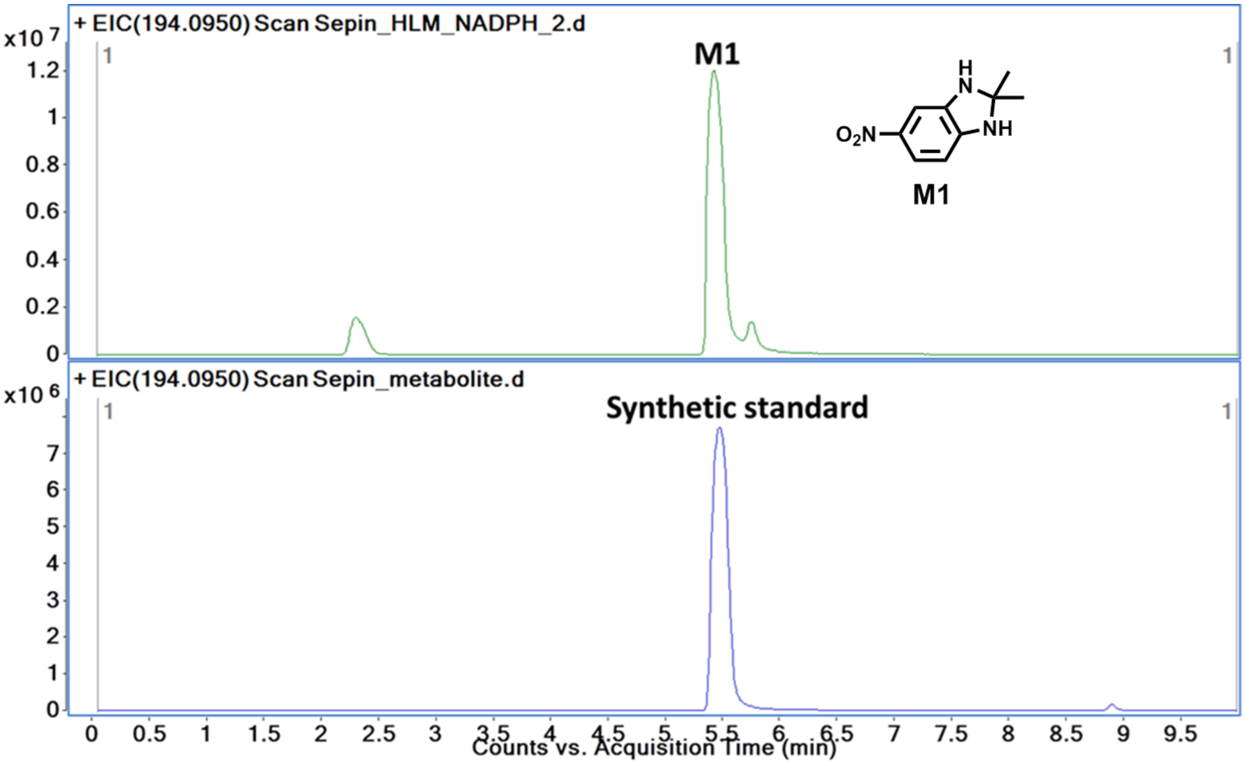

S_Table1. Summary of substrates, specific metabolites, transition ions, and positive control inhibitors used in the inhibitory experiments of sepin-1 on P450s.

Enzyme substrates	Specific metabolites	Precursor ion (m/z)	Product ion (m/z)	enzymes	Positive control inhibitor
Phenacetin	Acetaminophen	152.3	110.3	CYP1A2	α-Naphthoflavone
		152.3	65		
Efavirenz	8-O-Efavirenz	332.1	248.0	CYP2B6	Ticlopidine
		332.1	164.0		
Paclitaxel	6-O-Paclitaxel	892.3	308.1	CYP2C8	Quercetin
		892.3	286.1		
Diclofenac	4-O-Diclofenac	312.0	266.0	CYP2C9	Sulphaphenazole
		312.0	230.0		
(S)-Mephenytoin	4-O-(S)-Mephenyltoin	235.1	150.2	CYP2C19	Ticlopidine
		235.1	132.8		
Dextromethorphan	Dextromethorphan O-demethylation	257.9	200.6	CYP2D6	Quinidine
		257.9	157.1		
Midazolam	1-O-midazolam	342.2	203.2	CYP3A4	Ketoconazole
		342.2	324.1		
